# Hemolytic specimens in complete blood cell count: Red cell parameters could be revised by plasma free hemoglobin

**DOI:** 10.1002/jcla.23218

**Published:** 2020-01-22

**Authors:** Zhaoyang Peng, Wenqing Xiang, Jianming Zhou, Jiajia Cao, Zhe Li, Hui Gao, Junfeng Zhang, Hongqiang Shen

**Affiliations:** ^1^ Departments of Clinical Laboratory The Children's Hospital Zhejiang University School of Medicine National Clinical Research Center for Child Health Hangzhou China

**Keywords:** blood cell count, diagnostic errors, free hemoglobin, hemolysis

## Abstract

**Introduction:**

Hemolysis is the main cause of unqualified clinical samples. In this study, we established a method for detecting and evaluating hemolysis in whole blood test. We used a mathematical formula for correcting the influence of hemolysis on complete blood cell count (CBC) so as to avoid re‐venipuncture and obtain more accurate parameters of red blood cell detection, reduce the burden of patients, and improve the efficiency of diagnosis and treatment.

**Methods:**

Hemolytic samples were selected and then corrected using the new formula. Plasma free hemoglobin (fHB) was used as the criterion to determine the degree of hemolysis; the uncertainty of measurement is acceptable as the limit value of deviation between the measured value and the revised value. Hemolysis simulation analysis in vitro and continuous monitoring of clinical patients were used to verify the correction effect.

**Results:**

A total of 83 clinical samples with hemolysis were collected and analyzed; fHB 1.4 g/L was selected as the unacceptable value for clinical hemolysis detection. In hemolytic samples, the red blood cell parameters corrected by formula are significantly different from those uncorrected and had a good consistency with those before hemolysis.

**Conclusion:**

The results show that the hemolysis phenomenon of CBC has a significant impact on routine blood testing. By using the new formula, the influence of hemolysis on erythrocyte and related parameters can be quickly and easily corrected, thus avoiding venipuncture again for re‐examination, reducing diagnostic errors, and saving medical resources.

## 1 INTRODUCTION

Over the last decade, new methods for complete blood cell count (CBC) have been developed. Some of the novel technologies include automatic detection assembly line, cell image recognition and capability evolution based on neural network, and expert approval system based on logic tree. Still, an important foundational work has been excluded from the intelligent detection process, and that is the examination of sample traits.

The pre‐analysis stage mainly relays on the acquisition of qualified samples and the appropriate clinical test application, which is the premise of the test activities and an important basis for the test quality assurance.^1^ However, this stage is preformed outside the laboratory, involving more departments and a larger time and space span, thus might be prone to inspection errors.^2^, ^3^ The whole process monitoring of sample collection and transportation is highly efficient process, which includes standardization and automation; yet, the laboratories are often unable to achieve timely and comprehensive quality audit when receiving the samples, which often results in unqualified specimens before the start of the test or during the process. This extends the unqualified specimen turnaround time during the process of testing, which can easily cause the loss of reagents or equipment damage, and could even lead to medical errors.^3^, ^4^, ^5^

For CBC that uses anticoagulant blood, there is a greater possibility of unqualified conditions compared with the serum sample test items.^4^ Compared with blood coagulation, which can be observed by naked eye, microscopic blood agglutination can often be missed, thus seriously affecting the test results. Another common nonconformity that is commonly ignored in the field of CBC is the sample hemolysis.^6^, ^7^ Hemolysis is the most common problem observed during laboratory examination, which usually affects many test items.^8^, ^9^, ^10^, ^11^, ^12^ At present, biochemical tests have been applied as a standard for judging and grading the severity of hemolysis. They are based on different items and have different standard of acceptable degree of hemolysis.^13^, ^14^, ^15^ Today, many of the biochemical instruments relay on automatic identification and quantitative hemolysis sample capacity, which allows for the severity of detecting errors to be avoided.^16^, ^17^, ^18^, ^19^ However, because of the homogeneous sample, the detection of hemolysis in CBC is challenging, no matter which approach is used. It is thought that hemolysis is caused by damage to red blood cells, which can affect red blood cell count (RBC), mean corpuscular hemoglobin concentration (MCHC), and hematocrit (HCT), while the cell debris left from the broken red blood cells may also affect platelet count.

In this study, we used sample surveys to examine the incidence rate of hemolysis when preforming CBC. The severity of hemolysis was graded reasonably; the impact of hemolysis on the test project and the treatment countermeasures were analyzed, thus providing help for the comprehensive identification and treatment of hemolysis samples in the field of CBC.

## 2 MATERIALS AND METHODS

### 2.1 Materials

#### 2.1.1 Samples collection

This study was performed at the Department of Central Medical Laboratory, Children's Hospital Zhejiang University School of Medicine, Hangzhou, China. All blood samples were selected from discarded specimens and were collected from elbow vein or jugular vein in children and anticoagulated with K2‐EDTA. The clinical tests were completed within 2 hours after blood collection.

#### 2.1.2 Ethics

The Hospital Ethics Committee approved the study.

### 2.2 Instruments

Blood samples were tested on XN‐330 and XN‐A1 automatic blood analyzer (Sysmex). The reagents, calibrators, and quality controls were all from the same manufacturer. The instrument participated in national external quality assessment program of China Clinical Test Center and was in good status. Quality control measures were enforced to maintain instrument stability. Sample centrifugation was carried out on BY‐400C horizontal centrifuge (Beijing Baiyang Medical Instrument Factory).

### 2.3 Methods

#### 2.3.1 Collection and processing of clinical samples with hemolysis

The first study included venous blood samples that were received by the clinical hematology laboratory during 7 days in June 2018, workdays and weekends included. Briefly, samples with clotting or inadequate samples were excluded. Hemolyzed samples were manually selected by analyzing the red color in the supernatant plasma by naked eye, 1‐2 hours after standing. Some samples without visible hemolysis were used as control group to verify the centrifugation effect through the plasma free hemoglobin. The samples with hemolysis were mixed and then centrifuged using 800 *g* for 5 minutes. Plasma was separated, and the fHB was detected on sysmex XN‐L 330 blood analyzer. “Pre‐dilution” method was selected, in order to get more accurate results. The raw results were divided by seven to obtain the dilute coefficient 1:7. Then, the red blood cell parameters of the hemolyzed specimens could be revised by fHB, named as “fHB Revise Algorithm.” The procedure was performed using the following formula:

$$\text{Revised}\;\text{RBC}\;=\;\frac{\text{Hemolytic}\;\text{RBC}\;\times \;(\text{Hemolytic}\;\text{HB}\;+\;f\;\text{HB})}{\text{Hemolytic}\;\text{HB}}$$

$$\text{Revised}\;\text{MCHC}\;=\;\frac{\text{Hemolytoc}\;{\text{HB}}^{2}}{(\text{Hemolytic}\;\text{HB}\;+\;f\;\text{HB})\;\times \;\text{Hemolytic}\;\text{HCT}}$$

fHB, plasma free hemoglobin; Hemolytic RBC, red blood cell count of hemolyzed specimens; Hemolytic HB, hemoglobin of hemolyzed specimens; Hemolytic HCT, hematocrit of hemolyzed specimens; Revised RBC, red blood cell count of theoretical value generated by revise; Revised MCHC, mean corpuscular hemoglobin concentration of theoretical value generated by revise.

The significant difference between the actual measured value and the revised value was judged by the Uncertainty of Measurement, which was estimated according to CNAS‐TRL‐001:2012 “Medical Laboratory – Evaluate and Expression of the Uncertainty of Measurement.” Above all, the expanded uncertainty (U) and the combined standard uncertainty (u) of RBC and MCHC in this analyzer might be estimated. The bias of the actual measured value and the revised value was compared with the combined standard uncertainty, respectively, in RBC and MCHC; larger bias indicated significant difference between the two results in this analyzer.

#### 2.3.2 Hemolysis simulation in vitro

A total of 15 clinical surplus samples were selected for blood analysis. Artificial hemolysis resulted from mechanical damage to red blood cells, and it occurred when the syringe draw and detruded blood quickly for several times connected with needle. After stationary, the hemolysis phenomenon was confirmed for visual check; and then, blood analysis was performed. Then, fHB was determined according to the method aforementioned, and the corrected parameters were calculated. By comparing the erythrocyte parameters before and after hemolysis with the same sample, this method was used to verify whether the hemolysis correction formula can accurately reproduce the parameters of red blood cells before hemolysis.

#### 2.3.3 Continuous monitoring of clinical patients

A total of 5 patients from CICU who underwent multiple CBC testing during 5 days were selected, visible hemolysis occurred at least once during testing. By comparing the parameters of erythrocyte between non‐hemolytic samples and hemolytic samples before and after correction, we were able to verify the effect of our correction formula in clinical practice.

### 2.4 Statistical analysis

The paired *t* test was used to compare MCHC results between two groups. A *P*‐value < .05 indicated significant difference between the two groups.

## 3 RESULTS

A total of 3098 clinical blood samples were collected in the period of 1 week. Among those, 84 (2.71%) samples were hemolyzed. The source of hemolyzed samples covered almost all wards of the hospital; the top three were ICU (27, 32.1%), medical ward (19, 22%), and newborn ward (15, 17.86%). The plasma fHB was detected, after which the red cell parameters were revised by the formulas. The data distribution of the actual measured value and the revised value of some 84 hemolyzed samples is listed in Table 1.

**Table 1:** Data distribution of plasma fHB, detected values and revised values of hemolyzed samples

	Data distribution (CV ± SD)	Maximum	Minimum
fHB (g/L)	1.785 ± 1.149	6.6	0.3
Detected RBC (×10^12^/L)	4.376 ± 0.598	5.81	2.34
Revised RBC (×10^12^/L)	4.444 ± 0.610	5.85	2.4
Bias (%)	1.535 ± 1.060	6.3	0.23
Detected MCHC (g/L)	331.7 ± 11.9	361	294
Revised MCHC (g/L)	327.0 ± 13.3	358	282
Bias (%)	‐1.423 ± 1.094	2.48	−6.01

Laboratory reports that the uncertainty of measurement, the expanded uncertainty (U), and the combined standard uncertainty (u) of RBC and MCHC as follow: RBC: U = 2.01%, u = 1.01%; MCHC: U = 2.40%, u = 1.20%. When the detect bias over the uncertainty of measurement, the test results could not be accepted. Compared with the bias of the detected and revised results, the cutoff value was decided on plasma fHB ≥ 1.4 g/L with significant difference influenced by hemolysis of the red cell parameters.^20^, ^21^ The degree of hemolysis was defined with a visual assessment of supernatant plasma according to hemolyzed plasma color comparison card.^22^ For samples suspected to exceed the cutoff value, the final judgment results were determined by fHB detection. If they exceed the cutoff value, the formula “fHB Revise Algorithm” was used to correct the test results.

In the present study, 44 samples were judged as severe hemolysis (fHB ≥ 1.4 g/L). Among them, 34 patients underwent an additional CBC within 5 days. The MCHC of the check samples was used to evaluate the effect of correction. Significant difference was observed between the detected group (330.0 ± 13.3) and the revised group (322.8 ± 14.6) or checked group (323.7 ± 11.9) (all *P* < .01); while no significant differences were found between revised group and checked group (*P* = .3308).

**Figure 1:**
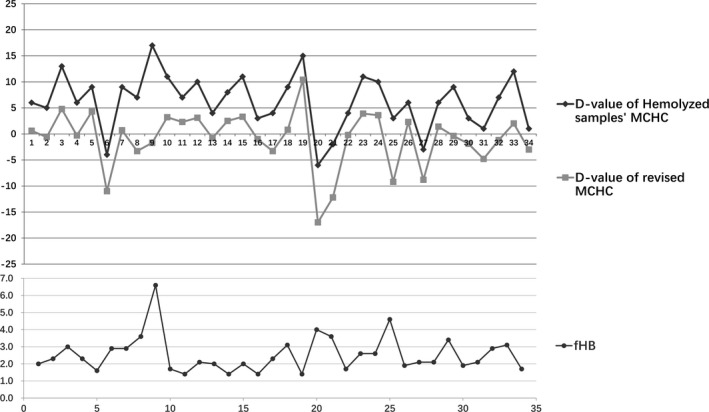
Bias of MCHC of hemolyzed samples and revised samples with check samples

The D‐values between hemolysis MCHC/revised MCHC and check sample MCHC are displayed in Figure 1. As expected, most of (39/44) revised MCHC results were accordant with check sample MCHC and were significantly different compared with the hemolysis MCHC. Five samples had higher check sample MCHC. Two out of 5 (No. 6 and 20) showed serious hemolysis and resulted in higher MCHC. Moreover, the patient No. 21, presented with high fever when the hemolyzed sample was collected, had recovered and was discharged without transfusion when the check sample was taken. The rise of check sample MCHC and whether it was influenced by disease and transfusion still remains unclear. The patient No. 27 was diagnosed as patent ductus arteriosus and had transcatheter closure on 27, June. The hemolyzed sample was collected on 25, June and the check sample on 29. We speculated that the heart blood flow returned to normal after surgery elevated hemoglobin and MCHC of the late check sample.

**Figure 2:**
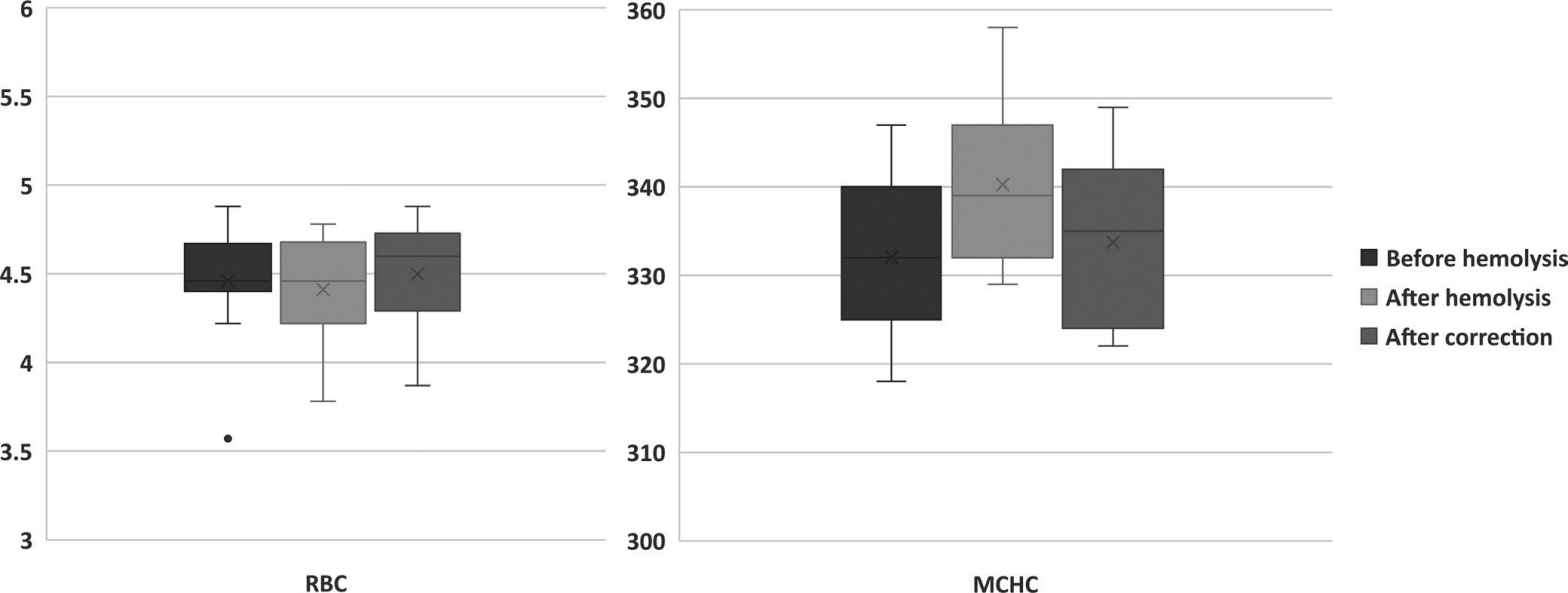
Comparison of hematological parameters before and after artificial hemolysis and after correction

In the second study, pre‐hemolysis parameters, post‐hemolysis parameters, and post‐hemolysis correction parameters of 15 hemolysis simulation samples were analyzed (Figure 2). The degree of artificial hemolysis was indicated by fHB (2.51 ± 0.76) in group after hemolysis. The MCHC significantly increased after hemolysis (332.1 ± 9.0, 340.3 ± 8.8, *P* < .01), while RBC and MCHC after formula correction (4.499 ± 0.286, 333.7 ± 8.8) were similar to that before hemolysis (4.461 ± 0.294, 332.1 ± 9.0, *P* > .05). In the 15 hemolysis simulation samples, abnormally elevated RBC after artificial hemolysis was observed in 6 samples. The results were incompatible with the destruction of red blood cells and the reduction of count numbers. Observation revealed a signification elevation at the end of platelet histogram in the six samples, where the means of the red cell fragments were so big that they were counted into red cells and the numbers were incorrectly elevated. Meanwhile, the platelet histograms were regularly in other samples.

**Figure 3:**
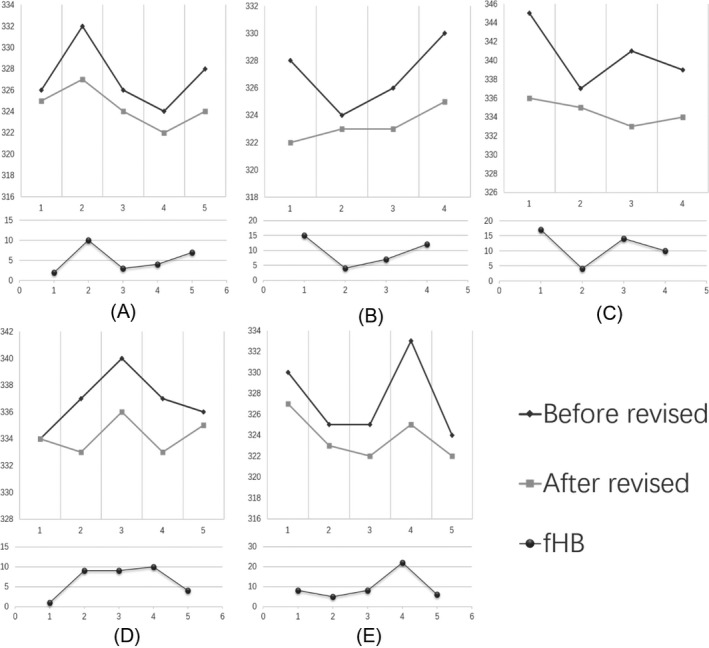
The MCHC of before or after revised in 5 patients

During the research period, in five patients from CICU with stable diseases, frequent CBC tests were performed and hemolysis (fHB ≥ 1.4 g/L) occurred at least once. Regardless of the degree of hemolysis, first, the red blood cell parameters from all the samples were revised by fHB one by one, and then, the measured MCHC and revised MCHC were used to make a comparison (Figure 3). This suggested that the fHB revise algorithm could regain the uniformity of the red blood cell parameters, such as MCHC.

## 4 DISCUSSION

A number of studies have shown that 70%‐80% of clinical decisions and subsequent efficacy assessments require the support of laboratory test data and diagnostic reports.^23^ And all clinical tests should profoundly rely on good quality control.^24^ Under evidence‐based medicine, any influencing factor that may lead to errors in clinical data and diagnostic reports can result in harm to patients,^25^ an increase in economic burden, a psychological injury, or even a threat to the lives of patients.^22^ Therefore, the importance of inspection quality assurance is much higher than the application and popularization of new projects and technologies. It is estimated that about 46.0%‐68.2% of the test results have unsatisfactory clinical feedback, which can ultimately be traced to insufficient sample quality.^3^ If the quality of the test specimens is not guaranteed, it is impossible to obtain accurate data, even though the follow‐up work is well preformed. Monitoring the unqualified rate of clinical samples is a very effective method of work. By performing data review and comparison over a fixed period, it is possible to identify the distribution of unqualified specimens in our laboratory, such as the main sources of clinical department, whether they were related to the patient group or individual medical staff, or to the sample flow in the hospital.^4^, ^26^, ^27^, ^28^

When preforming clinical CBC testing, we mainly consider factors that are easy to find, such as blood coagulation, and the mismatch of blood and anticoagulant, while hemolysis is often ignored as a potential problem. Hemolysis is the largest source of unqualified specimens in clinical practice.^4^, ^17^ As an anticoagulant and homogeneous test item, it is difficult to detect hemolysis in CBC timely and intuitively. Hemolysis usually appears due to mechanical compression during needle‐tube drawing, presence of certain physical or chemical foreign bodies which have been in contact with the blood, inappropriate anticoagulation or anticoagulant osmotic pressure mismatches, excessive oscillation in sample delivery or drastic changes in environmental conditions (such as temperature), excessive sample mixing before testing, etc Whatever the cause, hemolysis significantly affects CBC. For example, the broken red blood cells lead to a false reduction in the counting results, while the total hemoglobin content does not change, thus causing several other calculation sources of red blood cell parameters (such as MCH and MCHC) to result in deviation. In mild hemolysis, such deviations may not have a significant impact on the data's magnitude of the cause; nonetheless, in some cases, such as severe hemolysis or anemia, these deviations may mask the patient's pathological conditions, resulting in serious clinical consequences.^29^

Hemolysis does not affect the total hemoglobin in the blood sample. Thus, by detecting the free hemoglobin in the plasma, we can quantify the red blood cells affected by hemolysis. In this study, we have suggested a new correction formula, which can be used to correct the deviation caused by hemolysis to the parameters of red blood cells. In this study, we compared the corrected values of erythrocyte parameters of hemolytic samples with those of non‐hemolytic samples in the same patient. For a patient with stable condition, the change of MCHC was very small. As shown in Figure 3, the MCHC of the same patient after correction tends to be more consistent than before. The research conclusion proves that our calibration formula can effectively correct the deviation caused by hemolysis.

Our data suggested that hemolysis did affect the parameters of red blood cells. Free hemoglobin is used as an indicator of clinical acceptable cutoff value of hemolysis.^30^ The deviation of hemolytic samples below this value is not significant compared with the uncertainty of our laboratory testing system, that is, the accuracy of our laboratory testing system cannot well identify such mild hemolytic phenomena. The sample with hemolysis, which is higher than the cutoff value, has a significant effect on the parameters of the red blood cell. We believe that this cutoff value can be used as a criterion to classify the clinical acceptability of hemolytic samples on the premise that the uncertainty of the test items meets the clinical requirements, which can ensure that it will not have a serious impact on clinical diagnosis and treatment. It is suggested that each laboratory should evaluate the detection ability of hemolytic sample deviation in order to determine the cutoff value of clinical acceptability of hemolytic samples suitable for the laboratory, thus minimizing the impact of hemolysis on clinical test results.

**Figure 4:**
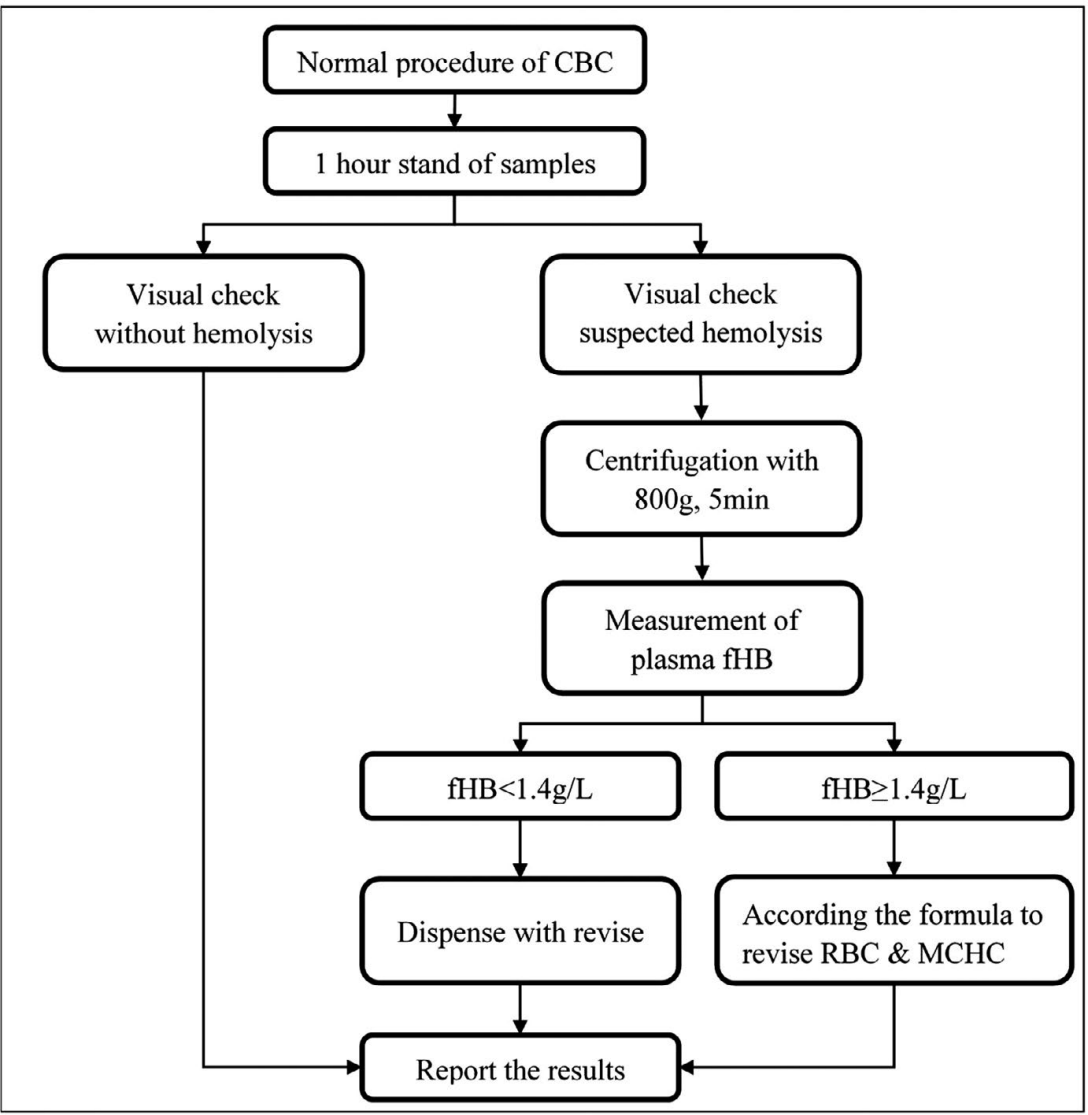
The processing flow of hemolysis CBC sample

To sum up, the laboratory needs to establish the processing flow of hemolysis CBC sample (Figure 4). Through this process, hemolytic samples can be found in a timely manner, and the severity of hemolysis can be assessed. At the same time, the impact of hemolysis can be corrected without the need for immediate re‐sampling, which can reduce the physical and psychological burden of patients while avoiding test errors.

## CONFLICT OF INTEREST

No potential conflicts of interest relevant to this article are reported.
